# AI-Assisted Simple Scoring Algorithm Was Helpful in the Risk Assessment of Cardiac Involvement in Patients with Pulmonary Sarcoidosis

**DOI:** 10.3390/jcm14207290

**Published:** 2025-10-15

**Authors:** Malgorzata Dybowska, Witold Z. Tomkowski, Katarzyna B. Lewandowska, Dorota Piotrowska-Kownacka, Malgorzata Sobiecka, Anna Kempisty, Lucyna Opoka, Piotr Radwan-Rohrenschef, Dorota Wyrostkiewicz, Monika Szturmowicz

**Affiliations:** 1 1st Department of Lung Diseases, National Tuberculosis and Lung Diseases Research Institute, Plocka 26, 01-138 Warsaw, Poland; w.tomkowski@igichp.edu.pl (W.Z.T.); k.lewandowska@igichp.edu.pl (K.B.L.); m.sobiecka@igichp.edu.pl (M.S.); annakempisty2@gmail.com (A.K.); parawan1970@wp.pl (P.R.-R.); dw707@wp.pl (D.W.); monika.szturmowicz@gmail.com (M.S.); 2 1st Department of Radiology, Medical University of Warsaw, 02-091 Warsaw, Poland; dorota.piotrowska-kownacka@wum.edu.pl; 3 Department of Radiology, National Research Institute of Tuberculosis and Lung Diseases, 01-138 Warsaw, Poland; lucyna.opoka@gmail.com

**Keywords:** cardiac sarcoidosis, cardiac magnetic resonance, artificial intelligence

## Abstract

**Background:** Cardiac involvement, one of the most life-threatening complications of sarcoidosis, remains under-recognized due to its oligo-symptomatic presentation in some patients. This retrospective study aimed to evaluate the utility of various clinical predictors of cardiac sarcoidosis (CS) development. **Methods:** The study included patients with pulmonary sarcoidosis diagnosed according to the recent ATS guidelines between January 2020 and July 2024 who underwent cardiac magnetic resonance (CMR) due to clinical suspicion of CS. The original Lake Louise criteria were used to identify active myocarditis. **Results:** Out of 393 patients diagnosed with pulmonary sarcoidosis, CMR was performed in 92 patients. Cardiac sarcoidosis was confirmed in 48 patients (52%, CS+), and excluded in 44 patients (48%, CS−). CS(+) patients demonstrated significantly more frequent Holter ECG abnormalities and liver/spleen sarcoidosis compared to CS(−) patients. Stage IV pulmonary disease, ECG abnormalities, and hypercalcemia were more common in CS(+) than in CS(−) patients; however, these differences did not reach statistical significance. Multivariate analysis identified Holter ECG abnormalities and liver/spleen involvement as significant predictive factors for CS, increasing the risk of cardiac involvement by approximately 4- and 6-fold, respectively. An AI-assisted simple scoring system based on five parameters: ECG abnormalities, Holter ECG abnormalities, liver/spleen involvement, gender, and stage of sarcoidosis predicted CS with a sensitivity of 76% and specificity of 74%, using an optimal cut-off value of ≥7.6 points. **Conclusions:** In patients with pulmonary sarcoidosis, an AI-assisted scoring algorithm derived from L1-regularized logistic regression results accurately predicted cardiac involvement on CMR with high specificity and sensitivity. Prospective validation of this algorithm is necessary to confirm its clinical utility in predicting cardiac sarcoidosis.

## 1. Introduction

Sarcoidosis is a heterogeneous, multi-organ systemic inflammatory disease of unknown etiology [1]. In genetically predisposed individuals, an exaggerated immune response to an unknown antigen promotes activation of type-1 T-helper cells (Th1) and subsequent secretion of inflammatory cytokines, such as interferon-γ (INF-γ), interleukin 2 (IL-2), interleukin 12 (IL-12), and tumor necrosis factor α (TNFα) [2]. This leads to the development of non-necrotizing granulomas in the affected organs, accompanied by varying degrees of lymphocytic inflammation [2].

Sarcoidosis most commonly affects the lungs and thoracic lymph nodes, but it can involve multiple organs, resulting in a wide range of clinical manifestations [1,3,4,5].

The incidence of sarcoidosis is influenced by several factors, including age, gender, ethnicity, and geographical location. The average incidence is estimated at 1–15 per 100,000; the highest rates are observed in Northern European countries [6]. Higher annual incidence of sarcoidosis was reported among African-Americans (35.5 per 100,000) [7]. Males predominate in the population diagnosed between 20 and 45 years of age (first peak of the disease), while females predominate in the second peak of the disease (50–60 years of age) [6].

Cardiac involvement is apparent at clinical presentation in 2–10% of unselected sarcoidosis patients [3,8]. Nevertheless, the true prevalence of cardiac sarcoidosis (CS) remains unknown, due to the oligosymptomatic course of the disease in some patients and a lack of agreement concerning the recommended diagnostic pathways [9]. On the other hand, cardiac involvement in sarcoidosis is associated with a poor prognosis. Patients who presented with clinical signs of CS had a 10% risk of sudden cardiac death over 5 years of follow-up [10].

In autopsy studies, cardiac involvement has been confirmed in 25–30% of sarcoidosis patients [11,12,13].

Thus, it is of high importance to develop the diagnostic algorithm for CS in both asymptomatic and symptomatic patients.

As an optimal screening algorithm for CS in sarcoidosis is still lacking, we conducted a single-center retrospective study to investigate the utility of various clinical predictors of CS and their combined value, based on an AI-assisted scoring algorithm.

## 2. Materials and Methods

### 2.1. Study Participants and Diagnosis for Pulmonary Sarcoidosis

We reviewed the source data of consecutive patients with pulmonary sarcoidosis (PS) hospitalized in a single pulmonary unit who underwent cardiac magnetic resonance (CMR) imaging between January 2020 and July 2024, due to suspected heart involvement.

According to recent ATS guidelines, pulmonary sarcoidosis was diagnosed based on the adopted criteria [14]:

1. Pathological evaluation (the finding of non-necrotizing granulomatous inflammation in one or more tissue samples) and exclusion of other granulomatous diseases;
2. Löfgren‘s syndrome (erythema nodosum, fever, arthritis, and bilateral hilar adenopathy with or without parenchymal disease) + spontaneous radiological regression;
3. Typical radiological features + hypercalcemia + renal failure.

### 2.2. Staging of Pulmonary Sarcoidosis and Diagnosis in Other Organs

Staging of pulmonary sarcoidosis was performed on the plain chest radiograph according to ATS and BTS guidelines and classified as follows: stage 0—normal radiograph; stage I—isolated hilar lymphadenopathy; stage II—hilar lymphadenopathy accompanied with parenchymal changes; stage III—parenchymal changes without hilar lymphadenopathy or pulmonary fibrosis; IV—lung fibrosis [14,15].

The diagnosis of sarcoidosis in other organs, such as the spleen/liver, was based on typical features on ultrasound or computed tomography (CT) imaging. Bone sarcoidosis was recognized based on CT and magnetic resonance imaging (MRI) presentation. Eye or skin involvement was confirmed by specialists in the appropriate medical disciplines.

The study was approved by the Ethics Review Board of our Institution (No. 13/2023).

### 2.3. Screening and Diagnostic Procedures of CS

At the time of PS recognition, all patients underwent a routine medical history review focused on symptoms such as dyspnea, chest pain, palpitations, or syncope, as well as blood tests including calcium levels, a 12-lead electrocardiogram (ECG), and echocardiography (ECHO). An echocardiographic examination was performed using a GE Vivid S70N ultrasound and a 2–5 MHz sector transducer (General Electric Company, Chicago, IL, USA).

Additionally, in patients with specific abnormalities in the 12-lead ECG or those complaining of arrhythmia, a 24 h Holter ECG examination was performed using the AsPEKT 703/712 device from ASPEL (Zabierzow, Poland).

In a 12-lead ECG, the presence of abnormalities such as intraventricular conduction disorders, supraventricular extrasystole (SVES), ventricular extrasystole (VES), supraventricular tachycardia (SVT), right axis deviation, and ST-T changes was noted.

Echocardiographic findings such as increased echogenicity of cardiac muscle, decreased left ventricular systolic function of less than 50%, right ventricular function measured by tricuspid annular plane systolic excursion (TAPSE), and the presence of pericardial effusion were assessed.

Significant rhythm disturbances in 24 h Holter ECG were defined as supraventricular extrasystole ≥ 200/24 h (SVES), ventricular extrasystole ≥ 200/24 h (VES), supraventricular tachycardia (SVT), non-sustained ventricular tachycardia (nsVT), ventricular fibrillation (VF), and idioventricular rhythm. Significant conduction disturbances were defined as sinus bradycardia < 30 BPM, 2nd-degree atrioventricular block (Wenckebach phenomenon), 3rd-degree atrioventricular block, and intraventricular conduction disorders with wide QRS.

**Figure 1:**
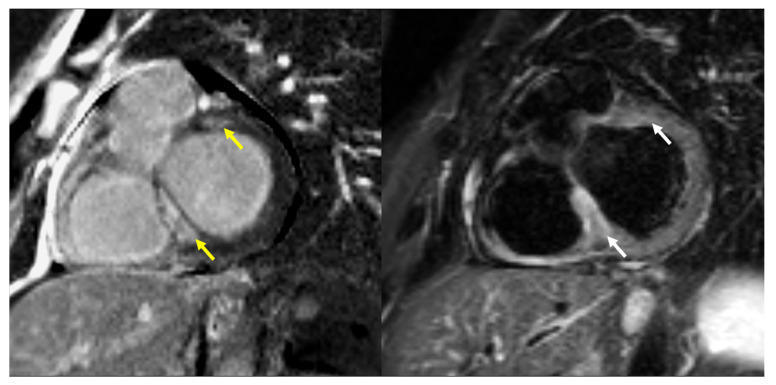
A 36-year-old male with pulmonary, cardiac, and splenic sarcoidosis. Intramural, nonischemic focal enhancement (yellow arrows) in the basal interventricular septum and anterior segment of the left ventricle (LGE short axis image on the left). Edema (white arrows) is present in a similar location on the corresponding T2W STIR short-axis image (on the right).

**Figure 2:**
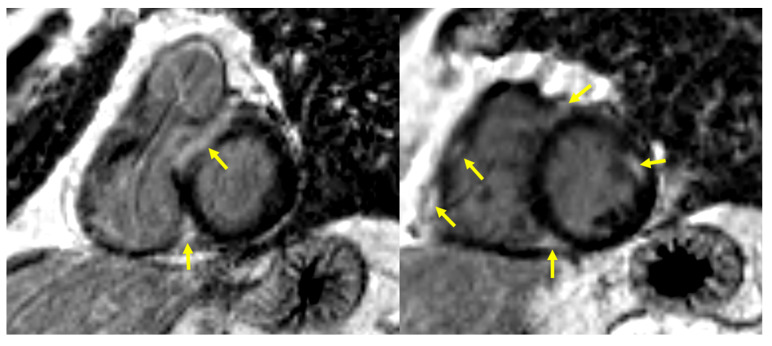
A 72-year-old male with pulmonary, cardiac, and splenic sarcoidosis. Multifocal, non-ischemic pattern of LGE (yellow arrows) in the interventricular septum, mid antero-lateral segment of the left ventricle and the right ventricular free wall—short axis post contrast images at the level of basal (on the left) and mid segments (on the right).

The decisive test for the diagnosis of cardiac involvement in sarcoidosis was cardiac magnetic resonance imaging (CMR). All examinations were performed on a commercially available 1.5 T scanner (Siemens Avanto, Erlangen, Germany) with a dedicated 32-channel cardiac/thoracic coil. The CMR protocol included evaluation of ventricular function, myocardial edema, and late gadolinium enhancement (LGE), all performed in corresponding short-axis and three long-axis views (two-, three-, and four-chamber). Following the original Lake Louise criteria, a diagnosis of active myocarditis was established when both myocardial edema and non-ischemic patterns of LGE were present [16]. Myocardial edema was evaluated on T2-weighted short tau inversion recovery (STIR) images. The presence of edema was confirmed if a focal area of hyperintensity was observed or if the ratio of left ventricular myocardial to skeletal muscle signal intensity on the same image exceeded 2. LGE was assessed on IR-prepared gradient echo images with the inversion time adjusted to null the signal from normal myocardium. A non-ischemic LGE pattern involving the interventricular septum, subepicardial or intramural regions, multifocal distributions, or the right ventricular free wall was considered positive for diagnosis (Figure 1 and Figure 2). Left ventricular systolic function analysis was performed with dedicated Argus software (Syngo MMWP VE27A workstation, Siemens, Erlangen, Germany). Systolic function parameters were provided; however, the results were not utilized for diagnostic purposes in cardiac sarcoidosis.

### 2.4. Statistical Analysis

The statistical analysis was performed using GraphPad Prism 10.4.2 (534) (GraphPad Software, LCC, San Diego, CA, USA). Continuous variables were presented as mean ± SD for normally distributed values or median and range for distributions that differed from normal. The t-test, the Mann–Whitney test, or the Wilcoxon test was used for between-group comparison in two groups, where appropriate. For the categorical variables, the numbers and percentages were presented, and the chi-square test or Fisher exact test were used to assess the differences. The *p*-value of <0.05 was considered statistically significant. Univariate and multiple logistic regression analyses were performed to determine the risk factors for the development of cardiac sarcoidosis.

L1-regularized logistic regression was performed to select significant predictors of cardiac sarcoidosis. Subsequently, ChatGPT (OpenAI, version 4o) was used as a computational assistant to convert regression coefficients into an integer-weighted, simplified score for clinical interpretability. A receiver-operated characteristic (ROC) curve was drawn for the new scoring system to determine its utility in the diagnostic algorithm. Full ChatGPT prompts are available in the Supplementary Materials.

## 3. Results

Between January 2020 and July 2024, 393 patients were diagnosed with pulmonary sarcoidosis. In 92 (23%), CMR was performed due to clinical suspicion of cardiac sarcoidosis. The group included 65 males and 27 females, with a mean age of 43.86 ± 10 years. Detailed characteristics of the examined group are presented in Table 1.

Cardiac involvement was confirmed in 48 out of 92 patients (52%). Clinical data of patients with cardiac sarcoidosis, CS(+), and those in whom cardiac sarcoidosis was excluded on CMR, CS(−), are presented in Table 1. No significant age differences were found between the groups. Males predominated in the entire study group and both subgroups, with a slightly higher percentage in CS(+) patients, compared to CS(−), 75% versus 66%, respectively. The majority of patients in both groups were diagnosed with stage II pulmonary sarcoidosis. Stage IV was diagnosed more frequently in CS(+) patients compared to CS(−) patients, at 17% and 7%, respectively, and stage I was diagnosed more frequently in CS(−) patients than in CS(+), at 25% and 6%, respectively. The differences in the distribution of different stages were of borderline significance (*p* = 0.0509). Liver and/or spleen involvement was found significantly more often in the CS(+) group compared to the CS(−) group, 27% vs. 4.5%, respectively, *p* = 0.0009. Hypercalcemia was present in 7 of 92 patients (8%), but it was seen almost exclusively in CS(+) patients (6 patients). Due to the small number of patients with hypercalcemia, these differences were not statistically significant.

The analysis of symptoms related to possible heart disease is presented in Table 2. Palpitations and/or syncope were noted in 29% of CS(+) patients and 18% of CS(−) patients; the difference was not statistically significant. In total, 35% of patients in CS(+) group and 36% in CS(−) group were asymptomatic.

The echocardiography results are presented in Table 3. The analysis included left ventricular ejection fraction (LVEF), increased echogenicity of cardiac muscle, TAPSE, and the presence of pericardial effusion. No significant differences were found between CS(+) and CS(−) groups concerning these parameters.

ECG and Holter ECG results are presented in Table 4. Abnormal ECG tracings were observed in 52% of CS(+) patients and 32% of CS(−) patients, *p* = 0.0594. Holter ECG revealed rhythm and/or conduction disturbances in 62% of CS(+) patients and 30% of CS(−) patients; this difference was highly significant (*p* = 0.009). The detailed analysis of the type of ECG and Holter ECG abnormalities is included in Supplementary Materials (Tables S1 and S2).

Univariate regression analysis revealed two factors significantly increasing the risk of cardiac involvement on CMR—abnormal Holter ECG (*p* = 0.008) and liver/spleen involvement (*p* = 0.017)—and two of borderline significance (*p* = 0.05–0.1)—ECG abnormalities and hypercalcemia. In multivariate analysis, abnormal Holter ECG and liver/spleen involvement were confirmed to be independent factors increasing the risk of cardiac sarcoidosis by four and six times, respectively (Table 5).

**Figure 3:**
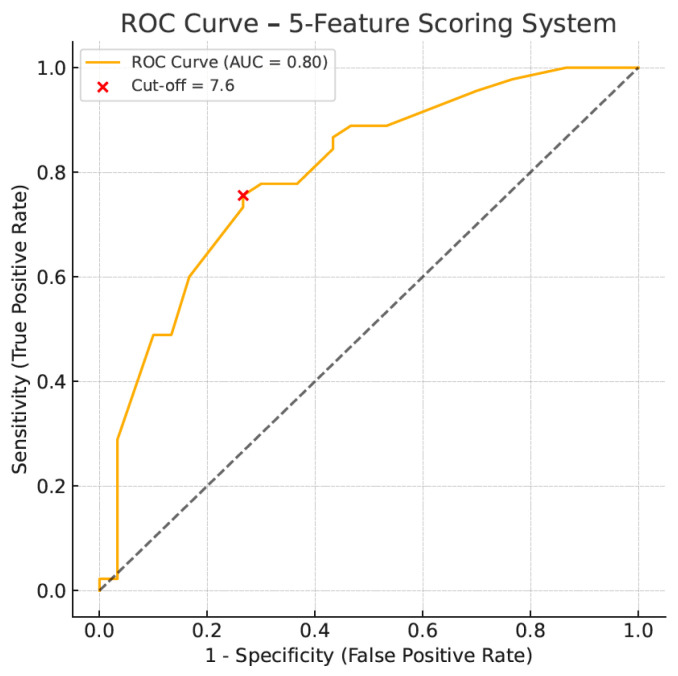
ROC curve for five-feature scoring system in the diagnosis of cardiac sarcoidosis.

**Figure 4:**
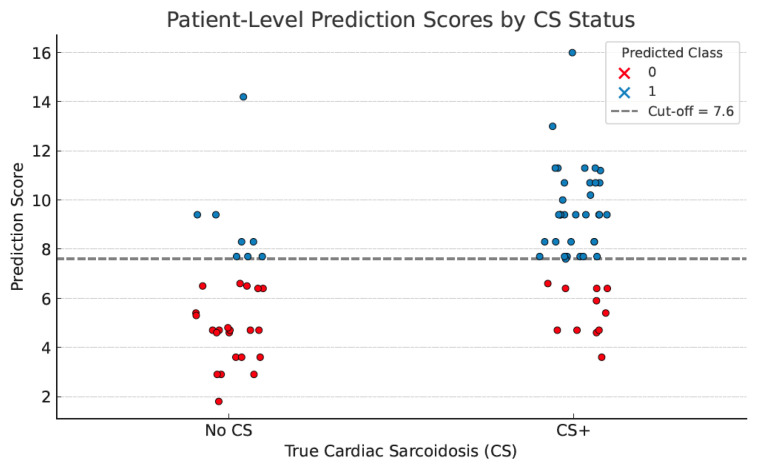
Distribution of prediction scores in CS(+) and CS(−) patients.

Based on L1-regularized logistic regression, eleven variables possibly predictive of cardiac sarcoidosis were analyzed, i.e., sex, age, sarcoidosis stage, Holter abnormalities, ECG abnormalities, ECHO LVEF, TAPSE, hypercalcemia, dyspnea, palpitations/syncope, liver/spleen (Supplementary Table S3). ECHO EF and TAPSE were excluded initially due to the null coefficient. Thereafter, five variables with the highest coefficient were selected with the assistance of AI, and a simplified scoring system was developed based on each weight for the prediction of the disease. It was defined as follows: score = 3.0 × (liver/spleen involvement) + 3.0 × (abnormal Holter ECG) + 1.8 × (sarcoidosis stage 1–4) + 1.8 × (abnormal ECG) + 1.1 × (sex, male-1, female-0). Each of the presented variables (except for sarcoidosis stage and sex) was coded as 1 if present or 0 if absent. The sarcoidosis stage was coded as follows: stage I—1 point; stage II—2 points; stage III—3 points; and stage IV—4 points. The optimal cut-off value was determined at ≥7.6 points. With this cut-off, CS could be predicted with a sensitivity of 76%, a specificity of 73%, a positive predictive value of 81%, and a negative predictive value of 67%. The ROC curve presenting the diagnostic utility of the simplified scoring algorithm is shown in Figure 3. The model demonstrated strong discriminatory power, AUC ROC 0.80. The plotted graph for each patient from the tested cohort is presented in Figure 4. The median score in patients with confirmed cardiac sarcoidosis was 8.3 points (95% CI, 7.88–9.36), whereas in patients without cardiac involvement, it was 5.1 points (95% CI, 4.87–6.77), *p* < 0.001. The diagnostic utility of the presented scoring algorithm exceeded that of any single parameter (Table 6).

## 4. Discussion

The diagnosis of cardiac involvement in sarcoidosis is difficult due to the oligosymptomatic and nonspecific presentation of the disease. Most clinicians use medical history and ECG as the first-line procedures to assess the probability of CS. According to the recent ATS recommendations, CMR should be used to establish the final diagnosis in all symptomatic patients or those with abnormal ECG results [14]. Such an approach may be combined with CMR overuse, increasing the costs of CS diagnostics. The other guidelines recommend echocardiography and/or Holter ECG as the second step in the CS diagnostic algorithm [14,17,18,19,20]. A recently published clinical consensus statement from the Heart Failure Association, the European Society for Cardiovascular Imaging, and the European Heart Rhythm Association Myocardial and Pericardial Diseases Working Group recommend further screening for CS with CMR in patients who present with at least one of the following: cardiac symptoms, elevated biomarkers, structural abnormalities, or arrhythmias [21]. Nevertheless, the optimal diagnostic approach for CS has not yet been defined.

In our study group, out of 92 patients with clinical suspicion of CS, CMR only confirmed active cardiac involvement in 48 (52%). Therefore, we attempted to find a more efficient screening algorithm based on demographic, clinical, electrocardiographic, and echocardiographic variables.

A male predominance was observed in the entire study group, with a slightly higher proportion in CS(+) patients compared to CS(−) patients, but the difference was insignificant. This was similar to the observation of Nakamura et al. [22], while Bakker et al. [23] and Martusewicz et al. [24] found significant male predominance in CS patients compared to non- CS ones. An interesting finding, presented by some researchers, is that CS in males often presents as ventricular arrhythmias, while in females, it presents as symptomatic heart failure [19].

None of the clinical symptoms that might suggest CS, such as palpitations/syncope, dyspnea, and chest pain, predicted cardiac involvement in our study group. CS(+) patients reported palpitations or syncope more frequently than CS(−) patients, at 29% vs. 18%, respectively; however, the difference was not statistically significant. Of great interest is that 35% of our patients with cardiac sarcoidosis confirmed on CMR were asymptomatic.

Heart Rhythm Study (HRS) screening recommendations included palpitations of more than two weeks ‘duration and a history of presyncope or syncope’, as symptoms indicative of CS. In contrast, modified clinical criteria used by Holzclaw et al. also included a history of non-pleuritic chest pain [17,18]. In patients with CS coexisting with lung involvement, symptoms may be related to heart involvement or progressive pulmonary disease [19]. According to the current British Thoracic Society (BTS) Clinical Statement, cardiac sarcoidosis should be considered in those patients with pulmonary sarcoidosis who present with dyspnea disproportionate to their lung function impairment [15].

Abnormal ECG results were observed in 52% of CS(+) patients and 32% of CS(−) patients in our group, with a borderline significant difference. However, the detailed analysis did not reveal any specific type of ECG disorders in CS(+) patients. According to the literature, the baseline electrocardiogram is abnormal in 7% of patients with sarcoidosis [14] and 90% of CS patients [25]. The most indicative of cardiac involvement are right bundle branch block; atrioventricular blocks of types I, II, or III; and ventricular arrhythmias including ventricular premature beats or ventricular tachycardia [25]. Atrial fibrillation episodes, ST-T changes, and abnormal Q waves, which reflect left ventricular muscle damage, may also be noted [25,26]. The diagnostic utility of ECG in CS screening depends on the definition of “abnormal ECG”. Most investigators agree that conduction abnormalities and/or ventricular ectopy are most suggestive of CS [14,17,18]. Pathologic Q waves present in more than two leads are frequently included in the definition [17,18,27]. Data on the diagnostic utility of the ECG for the recognition of CS analyzed by ATS experts are widely divergent, with reported sensitivity ranging from 9% to 92% and specificity of 73–97% [14]. In our study group, an abnormal ECG was 52% sensitive but only 68% specific as a method for predicting cardiac sarcoidosis.

Holter ECG monitoring displayed much higher diagnostic utility compared to ECG. Rhythm and/or conduction disturbances were noted in 62% of CS(+) and 30% of CS(−) patients. Detailed analysis revealed a significantly higher frequency of rhythm disturbances, particularly supraventricular extrasystole and supraventricular tachycardia, in CS(+) patients compared to those without CS(−). Non-sustained ventricular tachycardia and ventricular fibrillation were noted rarely, but exclusively in CS(+) patients. Abnormal Holter ECG was 62% sensitive and 70% specific for predicting CS, independently increasing the risk of cardiac involvement by four times. The results published by other authors are contradictory, showing the sensitivity of Holter ECG to be 59–67% and the specificity to be 21 to 95% [14,28,29]. Recently, Bakker et al. found that QRS > 120 ms and nsVT in Holter ECG were the signs predictive of CS and included this procedure in their screening algorithm [23]. Despite its potential diagnostic utility, routine Holter ECG use in the CS diagnostic algorithm was not recommended by the authors of the ATS guidelines [14]. BTS specialists suggest this test only for those sarcoidosis patients complaining of palpitations [15]. A recently described prospective observational study, CASPA for CS screening at Royal Papworth Hospital, included Holter ECG in the diagnostic pathway [29], but the results have not yet been published [30].

Due to its availability and non-invasive nature, standard 2D transthoracic echocardiography (TTE) is often the first choice among imaging tests in patients with suspected CS. However, TTE findings are frequently not sensitive enough for the early phase of CS [31]. Moreover, comparing different studies is challenging due to the lack of consensus regarding TTE criteria that suggest CS. HRS screening recommendations utilized the following TTE criteria: regional wall motion abnormalities, wall aneurysm, regional wall thinning, and left ventricular LVEF < 40% [18]. The other authors indicated basal septum thickening or wall thinning and LVEF < 50% as related to the cardiac sarcoidosis [17,30]. An increased echogenicity of the ventricular wall (bright aspect), localized particularly in the interventricular septum or left ventricular free wall, is often observed [31]. Rarely, echocardiographic signs of dilated or restrictive cardiomyopathy may be diagnosed [32,33,34,35]. Our TTE criteria included LVEF < 50 and focal hyperechogenicity of the ventricular wall. Additionally, we analyzed TAPSE and the presence of pericardial effusion. A local increase in echogenicity was observed in 56% of CS(+) patients and 43% of CS(−) patients. LVEF was comparable in both groups, and LVEF < 50% was a rare event noted in 8.3% of CS(+) and 4.3% of CS(−) patients. Applying the newer TTE techniques, such as Doppler, strain, and speckle tracking, may enhance the recognition of subclinical myocardial dysfunction in CS [33]. In particular, strain imaging improves TTE’s ability to detect myocardial involvement [33,34,35]. According to Murtagh et al., in patients with preserved LVEF, a decrease in left ventricular global longitudination strain exceeding 17%, displayed 94% sensitivity and specificity, in the recognition of CS [11]. Unfortunately, such modern techniques of TTE assessment were not available for the study group presented.

We also found that cardiac involvement was less frequent in stage I pulmonary sarcoidosis, and more frequent in stage IV (these differences were of borderline significance). In patients with liver and/or spleen involvement, the risk of CS was increased six times, which was the strongest predictor of CS in our study group. Bakker et al. found liver/spleen sarcoidosis to be more frequent in CS patients, but no information concerning the significance of stage IV pulmonary disease was noted [23]. The interesting and original finding in our study group was the coexistence of CS with hypercalcemia; out of seven patients with hypercalcemia, six also presented with cardiac sarcoidosis. This observation must be verified in a much larger group of patients, as hypercalcemia is rare in sarcoidosis. To the best of our knowledge, this is the first time such a coincidence has been reported.

In summary, the diagnostic utility in CS prediction in our study group was most pronounced for Holter ECG and liver/spleen involvement. Nevertheless, many other parameters could add to diagnostic performance. Thus, we used AI-supported analysis to find a more efficient diagnostic algorithm. The established scoring system was finally based on five parameters: liver/spleen involvement, abnormal Holter ECG, stage of pulmonary sarcoidosis, abnormal ECG, and gender. The strongest predictors were differentiated by the most significant coefficients. The median score in patients with confirmed cardiac sarcoidosis was 8.3 points (95% CI, 7.88–9.36), whereas in patients without cardiac involvement, it was 5.1 points (95% CI, 4.87–6.77), *p* < 0.001. The optimal cut-off value with the greatest discriminatory power was 7.6 points. With this cut-off, the algorithm could predict CS with 76% sensitivity, 73% specificity, 81% positive predictive value, and 67% negative predictive value.

## 5. Strengths of the Study

To our knowledge, this is the first attempt to utilize AI-assisted analysis in building a scoring system that assesses the probability of cardiac involvement in sarcoidosis. The presented scoring algorithm could predict CS on CMR with high sensitivity and specificity. Moreover, the system’s advantage lies in the application of simple parameters that are easy to assess in every sarcoidosis patient. The only modification from the recommended diagnostic procedures would be the increased implementation of Holter ECG monitoring. This tool could potentially reduce the need for costly CMR by identifying patients at lower risk, especially in resource-limited settings. It could be integrated into stepwise diagnostic algorithms and evaluated in future cost-effectiveness analyses. Nevertheless, we are aware that such a scoring system needs prospective validation on a much larger group of patients to determine its diagnostic utility.

## 6. Study Limitations

Our study had several limitations. First of all, this was a single–center study; therefore, the number of patients considered for CS presence, and those in whom CS was finally confirmed, was relatively low. On the other hand, cardiac involvement is not a very frequent localization in sarcoidosis, and we had to include 393 consecutive BBS patients to confirm cardiac sarcoidosis in 48 (12%). Secondly, due to the study’s retrospective character, the Holter ECG results were lacking in 17 out of 92 patients. We performed complete-case analysis only for the multivariate model; no imputation was applied. Thirdly, we did not use strain echocardiography in the presented study group; thus, contrary to recent publications, we could not demonstrate the diagnostic utility of echocardiography. Additionally, verification bias was present because CMR was performed only in patients with clinical suspicion of CS, which likely inflated diagnostic performance. Thus, our findings apply primarily to high-risk, CMR-referred populations. Furthermore, we used the 2009 Lake Louise criteria due to lack of access to T1/T2 mapping during the study period. The study protocol in all patients included LGE and T2STIR imaging, which are the basis of the 2009 criteria, but are also included in the criteria modified in 2018. The modified 2018 criteria allow for the diagnosis of acute myocarditis based on the presence of focal or diffuse fibrosis by LGE and/or extracellular volume mapping (ECV) as well as active inflammation using the following techniques: T2STIR, T1, or T2 mapping [36,37]. In our CMR unit at the time of the study assessments, we did not have access to mapping techniques. The use of the 2009 Lake Louise criteria is still justified by the ESC [21] and AHA recommendations [19]. The echocardiographic assessment in our study did not include GLS analysis, which is currently widely recognized as the most sensitive echocardiographic marker for subclinical myocardial dysfunction, including in cardiac involvement in sarcoidosis. The lack of GLS assessment undoubtedly constitutes a significant limitation of the study in the field of modern echocardiographic diagnosis of subclinical myocardial dysfunction. GLS echocardiographic analysis was unavailable in our retrospective dataset. Conclusions regarding echocardiographic assessment in the study were based on the above-mentioned echocardiographic parameters. Finally, due to small dataset size, we did not perform model comparisons—this is the aim for the future prospective analysis.

## Figures and Tables

**Table 1:** Characteristics of sarcoidosis patients with and without cardiac involvement according to CMR.

Parameter	Whole Group (N° 92)	Cardiac Involvement on CMR		*p*
		Yes (N° 48)	No (N° 44)	
Age, mean (±SD)	43.86 (±10.00)	43.04 (±10.6)	44.7 (±9.1)	0.416
Gender (N°, %)				0.368
M	65 (71)	36 (75)	29 (66)	
F	27 (29)	12 (25)	15 (34)	
Lung involvement				0.0509
Stage (N°,%)				
I	14 (15)	3 (6)	11 (25)	
II	60 (65)	34 (71)	26 (59)	
III	7 (8)	3 (6)	4 (9)	
IV	11 (12)	8 (17)	3 (7)	
Other organs, symptoms (N°, %)	25 (27)	17 (35)	8 (18)	0.063
Hypercalcemia	7 (8)	6 (13)	1 (2)	0.113
Liver, spleen	15 (16)	13 (27)	2 (4.5)	0.0009
Eye	2 (2)	0	2 (4.5)	0.437
Skin	2 (2)	1 (2)	1 (2)	0.514
Bones	1 (1)	1 (2)	0	0.96

**Table 2:** Symptoms related to possible heart disease in sarcoidosis patients according to cardiac involvement on CMR.

Parameter	Whole Group (N° 92)	Cardiac Involvement on CMR		*p*
		Yes (N° 48)	No (N° 44)	
Palpitations/syncope N° (%)	22 (24)	14 (29)	8 (18)	0.234
Chest pain N° (%)	25 (27)	11 (23)	14 (32)	0.359
Dyspnea N° (%)	32 (35)	19 (40)	13 (30)	0.383
No symptoms N° (%)	33 (36)	17 (35)	16 (36)	>0.999

**Table 3:** Echocardiography results in sarcoidosis patients according to cardiac involvement on CMR.

Parameter	Whole Group (N° 92)	Cardiac Involvement on CMR		*p*
		Yes (N° 48)	No (N° 44)	
LVEF%, mean (±SD)	58 (±8)	58 (±9.4)	58 (±6.3)	0.544
LVEF% < 50 N° (%)	6 (6.5)	4 (8.3)	2 (4.6)	0.679
TAPSE, mean (±SD)	23.74 (±3.09)	23.81 (±3.45)	23.66 (±2.68)	0.961
Increased echogenicity N° (%)	46 (50)	27 (56)	19 (43)	0.297
Pericardial effusion N° (%)	12 (13)	7 (15)	5 (11)	0.761

**Table 4:** ECG and Holter ECG results in sarcoidosis patients according to cardiac involvement on CMR.

Parameter		Whole Group	Cardiac Involvement on CMR		*p*
			Yes	No	
ECG	N°	92	48	44	0.0594
	Abnormal, N° (%)	39 (42)	25 (52)	14 (32)	
Holter ECG	N°	75	45	30	0.009
	Abnormal, N° (%)	37 (49)	28 (62)	9 (30)	

**Table 5:** Single and multiple logistic regression analysis of factors predictive of cardiac sarcoidosis.

	Single-Factor Analysis			Multiple Factor Analysis		
	OR	95% CI	*p*	OR	95% CI	*p*
age	0.9828	0.9420 to 1.024	0.412	1.034	0.9749 to 1.100	0.274
ECHO LVEF	0.9953	0.9446 to 1.048	0.855	1.039	0.9571 to 1.130	0.356
Holter ECG	**3.843**	**1.469 to 10.71**	**0.008**	**4.197**	**1.463 to 12.94**	**0.009**
Liver/spleen	**5.076**	**1.492 to 23.45**	**0.017**	**6.535**	**1.460 to 47.28**	**0.027**
ECG	**2.329**	**1.006 to 5.553**	**0.051**	1.931	0.6594 to 5.768	0.230
Hypercalcemia	**6.143**	**0.9913 to 118.6**	**0.099**	3.063	0.3770 to 65.08	0.348

**Table 6:** Diagnostic value of various parameters as predictors of cardiac involvement on CMR.

Parameter	Sensitivity	Specificity	PPV	NPV
Palpitations/syncope	29%	82%	64%	51%
Chest pain	23%	68%	44%	45%
Dyspnea	40%	71%	59%	52%
ECG	52%	68%	64%	57%
Holter ECG	62%	70%	76%	55%
Echocardiography—increased echogenicity	56%	57%	59%	54%
Echocardiography—increased echogenicity and/or decreased systolic function	69%	34%	53%	43%
**AI assisted scoring system**	**76%**	**73%**	**81%**	**67%**

## Data Availability

The datasets used and analyzed in this paper have been deposited in the National Tuberculosis and Lung Diseases Research Institute, Plocka 26, 01-138 Warsaw, Poland, and are available from the corresponding author on reasonable request.

## Supplementary Materials

The following supporting information can be downloaded at: https://www.mdpi.com/article/10.3390/jcm14207290/s1, Table S1. Detailed ECG abnormalities (Fisher’s exact test); Table S2. Detailed Holter ECG abnormalities (N = 75); Table S3. Variables predictive of cardiac sarcoidosis (L1-regularized logistic regression).

## Abbreviations

AI	artificial intelligence
ATS	American Thoracic Society
BPM	beats per minute
BTS	British Thoracic Society
CMR	cardiac magnetic resonance
CS	cardiac sarcoidosis
CS(+)	cardiac sarcoidosis conformed by cardiac magnetic resonance
CS(-)	cardiac sarcoidosis excluded on cardiac magnetic resonance
CT	computed tomography
ECG	electrocardiogram
ESC	European Society of Cardiology
ECV	extracellular volume mapping
GLS	global longitudinal strain
HRS	Heart Rhythm Study
IL-2	interleukin 2
IL-12	interleukin 12
INF-γ	interferon-γ (INF-γ)
JCS	Japanese Circulation Society
LGE	late gadolinium enhancement
LVEF	left ventricular ejection fraction
MRI	magnetic resonance imaging
nsVT	non-sustained ventricular tachycardia
PS	pulmonary sarcoidosis
ROC	receiver-operated characteristic
STIR	short tau inversion recovery
SVES	supraventricular extrasystole
SVT	supraventricular tachycardia
TAPSE	tricuspid annular plane systolic excursion
TNFα	tumor necrosis factor α
TTE	transthoracic echocardiography
VES	ventricular extrasystole
VF	ventricular fibrillation
