# Supplementary material for: AI-Assisted Simple Scoring Algorithm Was Helpful in the Risk Assessment of Cardiac Involvement in Patients with Pulmonary Sarcoidosis
Source: J Clin Med. 2025 Oct 15;14(20):7290. doi: 10.3390/jcm14207290 (PMC12564663; doi:10.3390/jcm14207290)
Supplement: Supplementary file 1 [file jcm-14-07290-s001.zip › Table S1.pdf]

Table S1.
Detailed ECG abnormalities (Fisher's exact test)

Type of abnormalities	CS+, N (%)	CS-, N (%)	p
ECG abnormalities (total)	25 (56)	14 (32)	0.0594
Intraventricular conduction disorders	10 (22)	9 (20)	>0.999
Supraventricular extrasystole (SVES)	2 (4)	2 (4)	>0.999
Supraventricular tachycardia (SVT)	2 (4)	0	0.4955
Ventricular extrasystole (VES)	3 (7)	3 (7)	>0.999
Right axis deviation	3 (7)	1 (2)	0.6181
ST-T abnormalities	7 (16)	3 (7)	0.3206
Normal tracings	23 (48)	30 (68)	
