# Supplementary material for: AI-Assisted Simple Scoring Algorithm Was Helpful in the Risk Assessment of Cardiac Involvement in Patients with Pulmonary Sarcoidosis
Source: J Clin Med. 2025 Oct 15;14(20):7290. doi: 10.3390/jcm14207290 (PMC12564663; doi:10.3390/jcm14207290)
Supplement: Supplementary file 1 [file jcm-14-07290-s001.zip › Table S2.pdf]

Table S2.

Detailed Holter ECG abnormalities (N=75)

Type of abnormalities	CS+, N (%)	CS-, N (%)	p
Conduction disturbances (any)	5 (11)	2 (7)	0.6952
- Sinus bradycardia <30 BPM	2 (4)	0	
- 2nd deg A-V block (Wenckebach)	1 (2)	2 (7)	
- 3rd deg A-V block	1 (2)	0	
- ICD with wide QRS	1 (2)	0	
Rhythm disturbances (any)	23 (51)	7 (23)	0.0183
- SVES ≥ 200/24 h	9 (20)	0	
- VES ≥ 200/24 h	10 (22)	5 (16)	
- SVT	5 (11)	2 (7)	
- nsVT	2 (4)	0	
- VF	1 (2)	0	
- Other*	3 (7)	0	
Normal tracings	17 (38)	21 (70)	

*idioventricular rhythm; ventricular beat pairs; supraventricular beat pairs
