# Supplementary material for: AI-Assisted Simple Scoring Algorithm Was Helpful in the Risk Assessment of Cardiac Involvement in Patients with Pulmonary Sarcoidosis
Source: J Clin Med. 2025 Oct 15;14(20):7290. doi: 10.3390/jcm14207290 (PMC12564663; doi:10.3390/jcm14207290)
Supplement: Supplementary file 1 [file jcm-14-07290-s001.zip › Table S3.pdf]

Table S3.
Variables predictive of cardiac sarcoidosis (L1-regularized logistic regression)

Feature	Coefficient
**Holter**	0.594
**Liver/Spleen**	0.591
**Stage (1–4)**	0.351
**ECG**	0.328
**Sex (M=1)**	0.222
Dyspnoea	0.187
Hypercalcaemia	0.090
Age	0.051
Palpitations	0.029
TAPSE	0
ECHO EF	0
